# Rapid Bacterial Detection and Gram-Identification Using Bacterially Activated, Macrophage-Membrane-Coated Nanowired-Si Surfaces in a Microfluidic Device

**DOI:** 10.1021/acs.nanolett.3c02686

**Published:** 2023-08-23

**Authors:** Sidi Liu, Huibo Wang, Le Yu, Yijin Ren, Hjalmar R. Bouma, Jian Liu, Henny C. van der Mei, Henk J. Busscher

**Affiliations:** † Institute of Functional Nano & Soft Materials (FUNSOM), Jiangsu Key Laboratory for Carbon-Based Functional Materials & Devices, Soochow University, 199 Ren’ai Road, Suzhou, 215123 Jiangsu P. R. China; ‡ University of Groningen and University Medical Center Groningen, Department of Biomedical Engineering, Antonius Deusinglaan 1, 9713 AV Groningen, The Netherlands; § University of Groningen and University Medical Center of Groningen, Department of Orthodontics, Hanzeplein 1, 9700 RB Groningen, The Netherlands; ∥ University of Groningen and University Medical Center Groningen, Department of Clinical Pharmacy and Pharmacology and Department of Internal Medicine, Hanzeplein 1, 9713 GZ Groningen, The Netherlands

**Keywords:** bacterial sepsis, extracorporeal blood cleansing, Gram-type, hemofiltration membranes, membrane fluidity, nanostructured surfaces

## Abstract

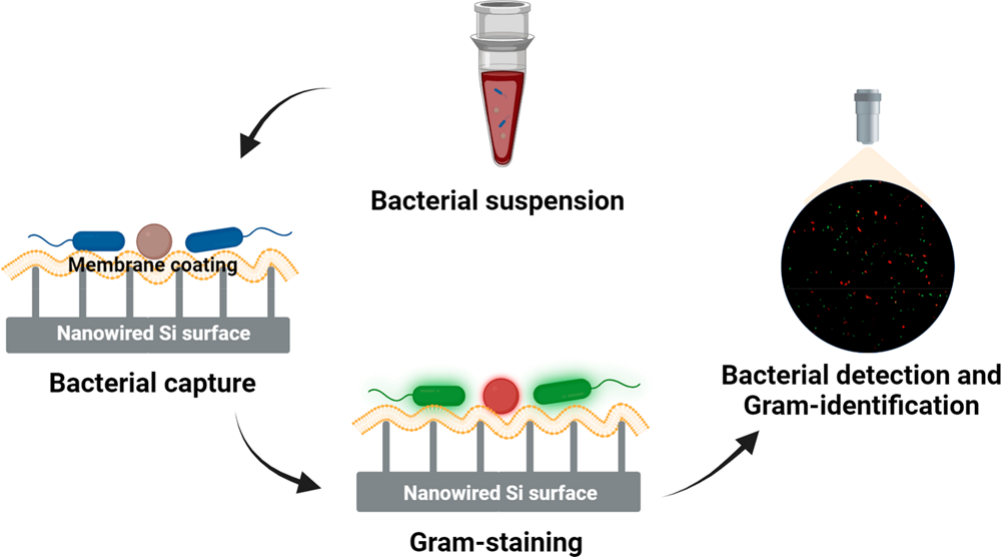

Bacterially induced sepsis requires rapid bacterial detection and identification. Hours count for critically ill septic patients, while current culture-based detection requires at least 10 h up to several days. Here, we apply a microfluidic device equipped with a bacterially activated, macrophage-membrane-coating on nanowired-Si adsorbent surfaces for rapid, bacterial detection and Gram-identification in bacterially contaminated blood. Perfusion of suspensions of Gram-negative or Gram-positive bacteria through a microfluidic device equipped with membrane-coated adsorbent surfaces detected low (<10 CFU/mL) bacterial levels. Subsequent, *in situ* fluorescence-staining yielded Gram-identification for guiding antibiotic selection. In mixed *Escherichia coli* and *Staphylococcus aureus* suspensions, Gram-negative and Gram-positive bacteria were detected in the same ratios as those fixed in suspension. Results were validated with a 100% correct score by blinded evaluation (two observers) of 15 human blood samples, spiked with widely different bacterial strains or combinations of strains, demonstrating the potential of the platform for rapid (1.5 h in total) diagnosis of bacterial sepsis.

Rapid bacterial elimination is extremely important in the treatment of bacterially induced sepsis to avert the development of organ failure and mortality.^1,2^ With the risk of death increasing by 7.6% every hour that initiation of treatment is delayed, it is clear that currently applied diagnosis based on blood-culturing that can take at least 10 h to several days requires more time than clinically available for therapeutic decision making.^3−5^ Preliminary infection control without any identification of the causative bacterial pathogen therefore necessarily involves broad-spectrum antibiotic administration that may or may not be effective in bacterial pathogen elimination from blood.^6,7^ Also the healthy microflora of critically ill patients may be compromised by broad-spectrum antibiotic administration, which is highly undesirable considering the condition of most septic patients.^8^

The great clinical relevance of the rapid diagnosis and differentiation of bacterial species has stimulated significant research in the field. Among the current research attempts,^5^ genomic sequencing and metagenomics require a high level of expertise while the time involved is high ranging from several days to weeks. Polymerase chain reaction techniques require several hours to days at a moderate level of expertise. Mass spectrometry requires minutes to a few hours for sample preparation and analysis and is also at a moderate level of expertise. However, due to the high costs, specialized training requirements, and the time involved in a variety of these methods, culturing has hitherto remained the clinical standard, for detection and identification, although the time required for culturing delays critical therapeutic decisions.^5^

Extracorporeal blood cleansing using hemofiltration membranes in microfluidic devices is another emerging therapy for blood cleansing in septic patients, although clinical benefits of hemofiltration are not convincing^9−11^ and novel adsorbent surfaces based on cell membrane coatings are required.^12^ Microfluidic platforms equipped with improved adsorbent surfaces, however, can be applied not only therapeutically but also diagnostically. Here, we demonstrate the use of a microfluidic device equipped with a bacterially activated macrophage membrane coating on nanowired-Si surfaces (see Figure S1 for architecture) for the rapid detection of bacteria in bacterially contaminated blood and their Gram-identification as a first criterion for antibiotic selection. To evaluate the possible diagnostic merits of this microfluidic platform, 1 mL of phosphate buffered saline (PBS) supplemented with different concentrations of Gram-negative *Escherichia coli* and/or Gram-positive *Staphylococcus aureus* was perfused through a microfluidic device, as outlined in Scheme 1. Concentration ranges were chosen to encompass low concentrations representative of clinically infected blood (1 to 10^2^ CFU/mL)^13,14^ to determine the detection limit of the platform as well as high concentrations up to 10^9^ CFU/mL in order to explore a possible upper limit of bacterial capture. In order to validate the results for clinical use, a blinded evaluation of 15 human blood samples spiked with different bacterial pathogens at different concentrations and a combination of pathogenic strains was carried out.

**Scheme 1:**
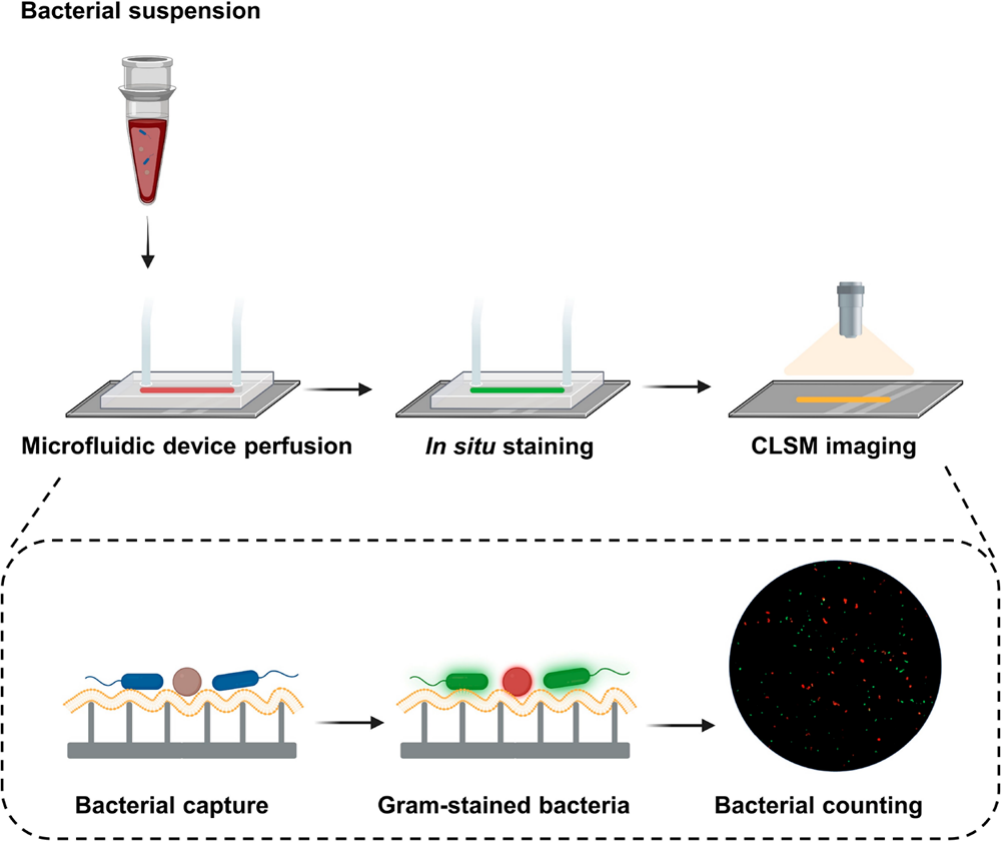
Bacterial Suspension Prepared with Gram-Positive and/or Gram-Negative Pathogens (Either in PBS or Blood) Perfused through a Microfluidic Device Equipped with an *E. coli* Activated, J774A.1 Macrophage-Membrane Coated, Nanowired-Si Surface as a Bottom Plate, Serving as an Adsorbent Surface After perfusing 1 mL of a bacterial suspension through the device at a flow rate of 1 mL/h, captured bacteria were stained inside the microfluidic device by a fluorescent Gram-stain (LIVE Baclight bacterial gram-stain kit) in water to identify Gram-negative, green-fluorescent or Gram-positive, red-fluorescent bacteria. Finally, Gram-positive and/or Gram-negative bacteria are enumerated using confocal laser scanning microscopy (CLSM).

The number of Gram-stainable *S. aureus* and *E. coli* captured increased sigmoidally with concentration for concentrations above 1 × 10^2^ CFU/mL (Figure 1), while leveling off above 1 × 10^7^ CFU/mL at a level of around 1 × 10^4^ CFU/cm^2^. In order to determine the detection limit of the platform, data for the lower concentrations of bacteria in suspension, representative for clinical sepsis,^13,14^ are also presented as a function of the number of CFUs per field of view. For the lower concentrations applied, five out of five experiments showed at least 1 bacterium within the microscopic field of view applied for a bacterial concentration in PBS of 10 CFU/mL, regardless of the strain considered. Accordingly, the detection limit for diagnostic use of this microfluidic platform under the conditions applied amounts to 10 CFU/mL. Bacterial detection of such low numbers, including Gram-identification, is achieved within 1.5 h, i.e., 1 h for perfusion through a microfluidic device and approximately 0.5 h for microscopic imaging. Obtaining similar information using culturing would require at least 10 h.^15^

**Figure 1:**
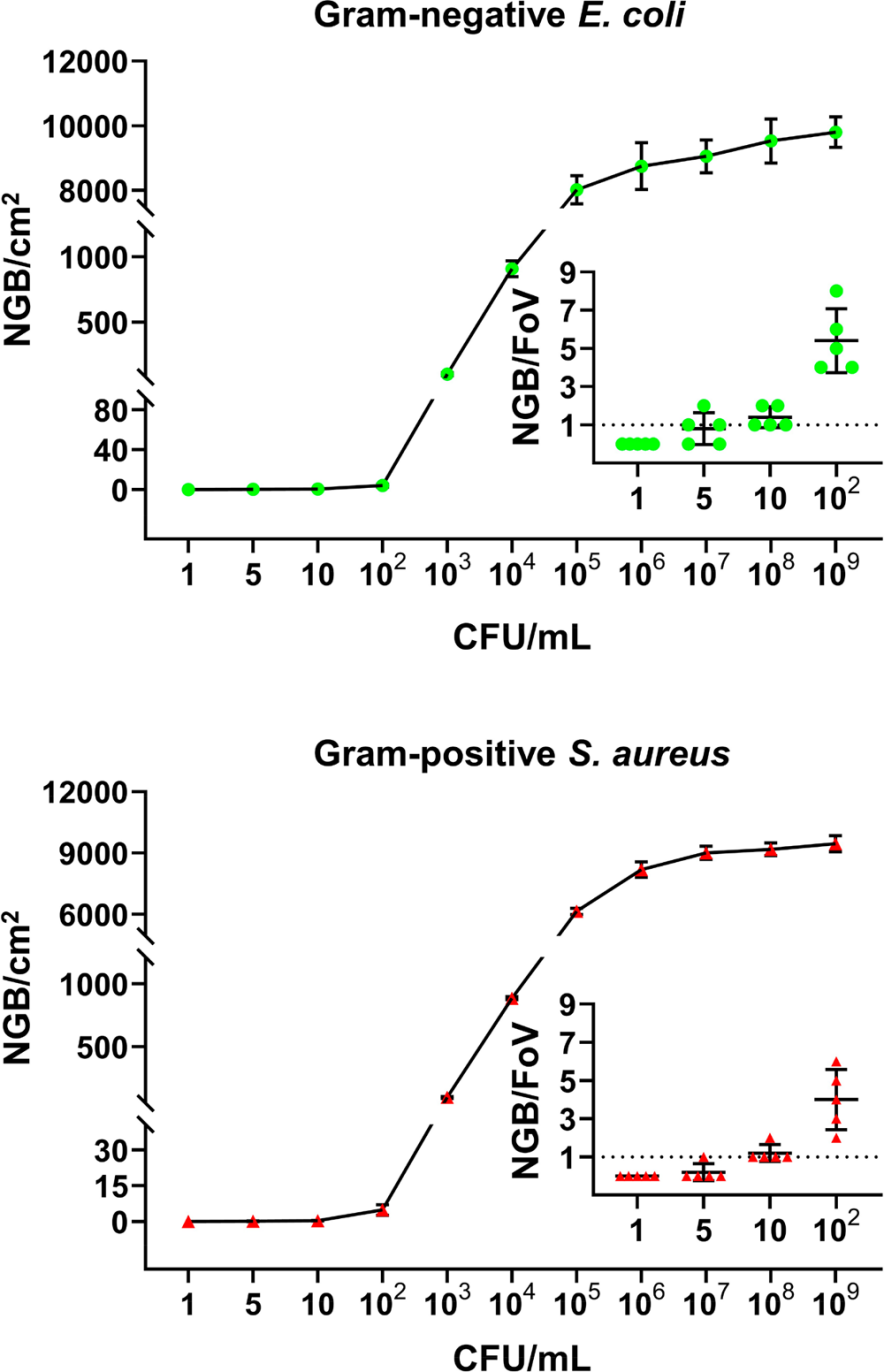
Number of Gram-stainable *E. coli* ATCC 25922 and *S. aureus* ATCC 12600 (NGB/cm^2^) captured per cm^2^ adsorbent surface as a function of the number of CFU/mL in PBS (NaCl 0.137 M, KCl 0.0027 M, Na_2_HPO_4_ 0.01 M, KH_2_PO_4_ 0.0018 M, pH 7.4) perfused through a microfluidic device. Error bars denote standard deviations over five different microfluidic devices perfused with differently prepared bacterial suspensions. Insets represent the number of Gram-stainable bacteria captured per microscopic field of view (FoV = 0.018 mm^2^ at a microscope magnification of 400 × ) as a function of the number of CFU/mL for concentrations up to 10^2^ CFU/mL. For each concentration, individual results from each experiment are given while the dashed line indicates the detection limit.

Since sepsis can also be the result of a combination of Gram-negative and Gram-positive bacterial pathogens, mixed suspensions of *E. coli* and *S. aureus* in different ratios were perfused through our microfluidic device. Fluorescent Gram-staining (see images in Figure 2A) allowed identification of the capture of *E. coli* and *S. aureus* from mixed suspensions in the same ratios as fixed in suspension (Figure 2B).

**Figure 2:**
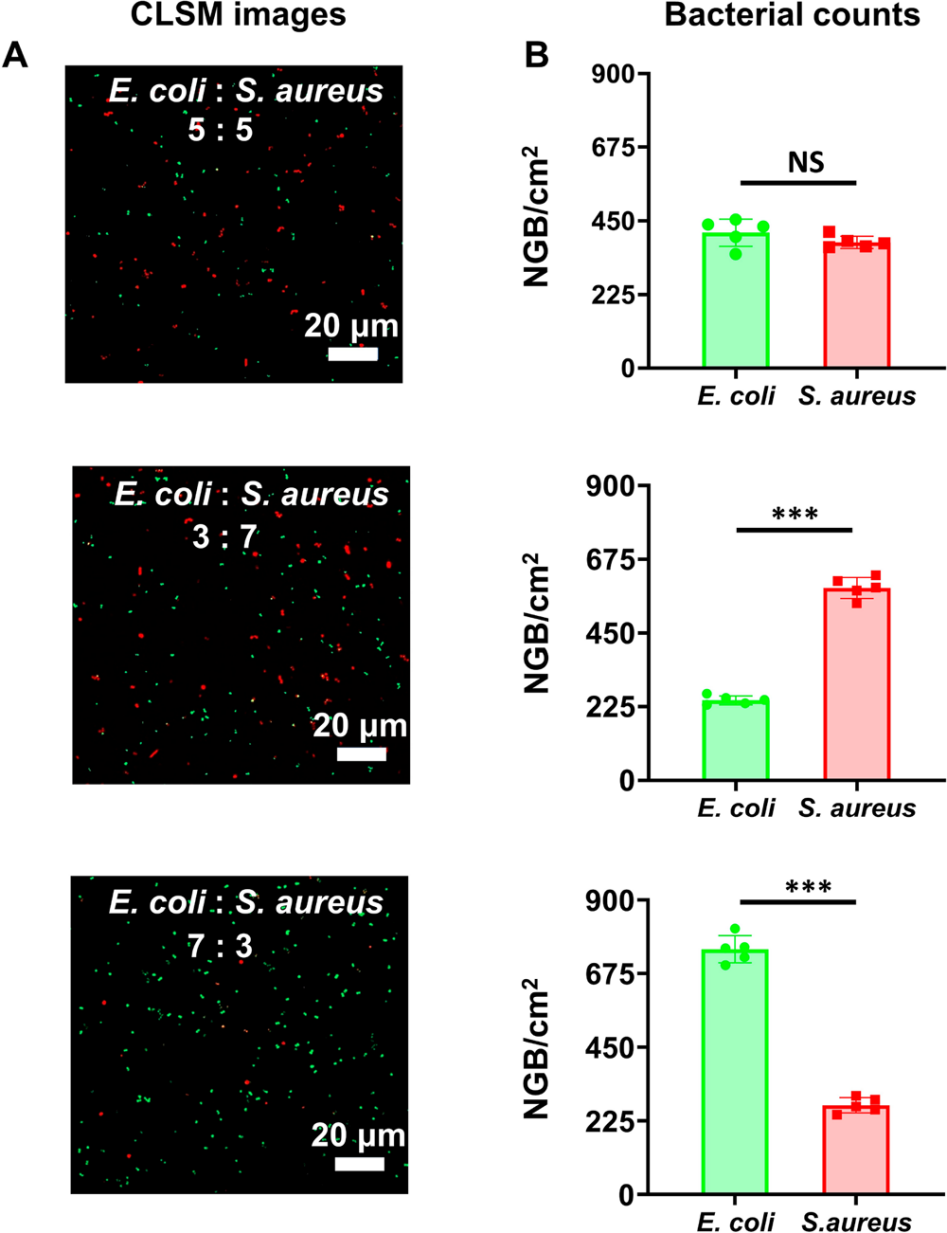
Capture of Gram-negative *E. coli* ATCC 25922 and Gram-positive *S. aureus* ATCC 12600 from mixed bacterial suspensions in PBS after perfusion through a microfluidic device equipped with an *E. coli*-activated, macrophage membrane-coated nanowired-Si bottom plate as an adsorbent surface. *E. coli* and *S. aureus* were mixed in different ratios. (A) CLSM images show captured green-fluorescent *E. coli* and red-fluorescent *S. aureus* mixed in suspension at different ratios. (B) The number of Gram-negative *E. coli* and Gram-positive *S. aureus* captured per cm^2^ adsorbent surface (NGB/cm^2^) from mixed *E. coli* and *S. aureus* suspensions. Error bars denote standard deviations over five different microfluidic devices, while asterisks indicate statistical significance (two-tailed *t* test) between the data indicated by the spanning bars (***p < 0.001, NS means no statistical significance).

Finally, the microfluidic platform as described was employed to detect and identify the presence and Gram-type in human blood spiked with widely different strains and combinations of strains within the clinically relevant concentration range up to 200 CFU/mL. Six commonly occurring bacterial strains in sepsis were used for spiking (see Table 1). Strains, their combinations, and concentrations were randomly computer-generated and analyzed by two blinded observers. A correct diagnosis was obtained within 1.5 h in 15 out of 15 samples, both with respect to bacterial presence and their Gram-type(s) and without any discrepancy between different blinded observers.

**Table 1:** Blinded Evaluation of 1 mL of Bacterially Spiked Human Blood Obtained from Healthy Donors^a^

	sample description			correct evaluation	
sample number	strain	Gram-type	concentration (CFU/mL)	of strain	of Gram-type
1	*Escherichia coli*	negative	100	yes	yes
	*Staphylococcus aureus*	positive	50	yes	yes
2	no spiking	no bacteria	0	yes	yes
3	*Klebsiella pneumoniae*	negative	50	yes	yes
	*Escherichia coli*	negative	100	yes	yes
4	*Enterococcus faecalis*	positive	20	yes	yes
	*Escherichia coli*	negative	100	yes	yes
5	*Staphylococcus aureus*	positive	50	yes	yes
	*Klebsiella pneumoniae*	negative	50	yes	yes
6	*Enterococcus faecium*	positive	20	yes	yes
	*Klebsiella pneumoniae*	negative	50	yes	yes
7	*Escherichia coli*	negative	100	yes	yes
8	*Streptococcus pneumoniae*	positive	20	yes	yes
	*Klebsiella pneumoniae*	negative	50	yes	yes
9	*Streptococcus pneumoniae*	positive	20	yes	yes
	*Staphylococcus aureus*	positive	50	yes	yes
10	no spiking	no bacteria	0	yes	yes
11	*Staphylococcus aureus*	positive	50	yes	yes
12	No spiking	no bacteria	0	yes	yes
13	*Enterococcus faecium*	positive	100	yes	yes
	*Escherichia coli*	negative	100	yes	yes
14	*Enterococcus faecium*	positive	20	yes	yes
	*Escherichia coli*	negative	100	yes	yes
15	*Klebsiella pneumoniae*	negative	50	yes	yes
	*Staphylococcus aureus*	positive	50	yes	yes

a Sample numbers, bacterial strains, combination of strains, and concentration within the clinically relevant range were randomly computer-generated, while experiments were carried out by two blinded observers with no access to the sample descriptions.

With the current application of 1 mL of blood perfusing through the device, correct diagnosis of infecting strains and their Gram-type could be obtained at a detection limit of 10 CFU/mL. Larger sample volumes and longer perfusion times may further lower the detection limit. With the current use, the entire diagnostic procedure can be carried out within 1.5 h, and the Gram-identification provides a first criterion for suitable antibiotic selection—including the decision not to prescribe antibiotics at all. Importantly, the diagnostic procedure could also be carried out in an extracorporeal circuit. This offers the advantage that, within the first 1.5 h of connecting a patient to a microfluidic device, diagnosis is performed based on low-level bacterial capture from blood. Since the upper limit for bacterial capture is significantly higher, this enables the further capture of bacterial pathogens for the (possibly necessary) simultaneous cleansing of blood.^12^ Therewith diagnosis and therapy, if required, are done at the same time, which may provide a life-saving strategy in critically ill patients.

The versatile detection and removal of widely different bacterial strains by bacterially activated macrophage membranes are achieved by bacterially activated macrophage membrane attachment to a nanowired-Si adsorbent surface (see also Figure S1). The nanowires provide small, isolated contact points between which the membrane is spanned. This leaves the major part of the membrane in contact with a fluid phase (Figure 3), allowing transmembrane proteins to float freely within the lipid membrane and rearrange into domains to form different toll-like and other types of receptors according to need, as in natural macrophage membranes to capture bacteria, PAMPs and excess cytokines. The natural blood compatibility of macrophage cell membranes ensures that these domains are not shielded with proteins adsorbed from a protein-laden fluid such as blood and remain to capture bacterial pathogens.

**Figure 3:**
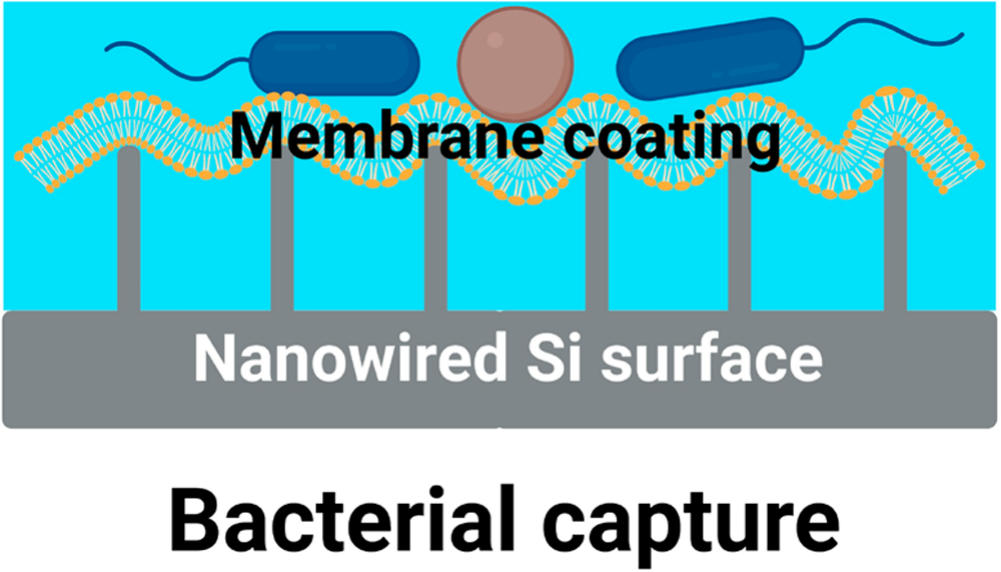
Capture of various Gram-negative and Gram-positive bacterial pathogens is achieved by virtue of the high fluidity of the membrane, spanned between small, isolated contact points on nanowired-Si surfaces. This leaves a dual-sided contact of the membrane with a fluid phase, essential for maintaining fluidity.

In summary, a microfluidic platform based on the use of bacterially activated, macrophage-membrane-coated nanowired-Si surfaces, as described in this paper, offers two major improvements with respect to the current state of research in the diagnosis of sepsis. First, it allows rapid detection (1.5 h) of extremely low numbers of bacteria from blood, while second, it simultaneously identifies the Gram-type(s) of the bacteria captured using fluorescent Gram-staining. This procedure may be life-saving, buying precious time for therapeutic decisions in the “golden critical hour”^16,17^ available to septic patients. Importantly, it can support the important therapeutic decision to administer antibiotics effective to Gram-positive or Gram-negative bacterial species without overly compromising the healthy microflora of a patient or totally withholding antibiotics, which is highly relevant in preventing a further rise in antimicrobial resistance.

## Supporting Information Available

Additional methods and figures including the architecture of nanowired-Si surfaces (PDF)
