# Supplementary material for: Rapid Bacterial Detection and Gram-Identification Using Bacterially Activated, Macrophage-Membrane-Coated Nanowired-Si Surfaces in a Microfluidic Device
Source: Nano Lett. 2023 Aug 23;23(17):8326–30. doi: 10.1021/acs.nanolett.3c02686 (PMC10510579; doi:10.1021/acs.nanolett.3c02686)
Supplement: Supplementary file 1 — nl3c02686_si_001.pdf [file nl3c02686_si_001.pdf]

Supporting information

Rapid Bacterial Detection and Gram-Identification using Bacterially-Activated, Macrophage Membrane-Coated Nanowired-Si Surfaces in a Microfluidic Device

Sidi Liu[a,b], Huibo Wang[a], Le Yu[a], Yijin Ren[c], Hjalmar R. Bouma[d], Jian Liu*[a], Henny C. van der Mei*[b] and Henk J. Busscher*[b]

[a]Institute of Functional Nano & Soft Materials (FUNSOM), Jiangsu Key Laboratory for Carbon-Based Functional Materials & Devices, Soochow University, 199 Ren'ai Rd, Suzhou 215123, Jiangsu, P. R. China.

Email: jliu@suda.edu.cn

[b]University of Groningen and University Medical Center Groningen, Department of Biomedical Engineering, Antonius Deusinglaan 1, 9713 AV Groningen, The Netherlands.

Email: h.c.van.der.mei@umcg.nl; h.j.busscher@umcg.nl

[c]University of Groningen and University Medical Center of Groningen, Department of Orthodontics, Hanzeplein 1, 9700 RB, Groningen, The Netherlands

[d]University of Groningen and University Medical Center Groningen, Department of Clinical Pharmacy and Pharmacology and Department of Internal Medicine, Hanzeplein 1, 9713 GZ Groningen, The Netherlands

* Corresponding authors

METHODS

***Bacterial culture conditions and harvesting*:**

Staphylococcus aureus ATCC 12600, *Streptococcus pneumoniae* ATCC 49619 *Enterococcus faecium* ATCC 35667 and *Enterococcus faecalis* ATCC 19433 (American Type Culture Collection, USA) were cultured on Tryptone Soy agar plates (HangZhou Microbial Reagent Co. LTD, China). *Escherichia coli* ATCC 25922 and *Klebsiella pneumoniae* CMCC(B) 46117 (National Center for Medical Culture Collection, China) were cultured on Luria-Bertani agar plates (HangZhou Microbial Reagent Co. LTD, China). One colony was inoculated in 10 mL of the appropriate liquid growth medium (Tryptone Soy Broth or Luria-Bertani) and incubated for 24 h at 37°C. This pre-culture was inoculated (1:200) into 200 mL fresh growth medium and incubated for 16 h. Bacteria were harvested by centrifugation (5000 ×g, 5 min) and washed twice with phosphate buffered saline (PBS; NaCl 0.137 M, KCl 0.0027 M, Na_2HPO_4 0.01 M, KH_2PO_4 0.0018 M, pH 7.4). Bacterial suspensions were diluted in PBS or growth medium to the concentrations required for the specific experiments. The final concentrations were determined using plate counting.

***Fabrication of a microfluidic device*:**

The microfluidic device was fabricated as previously reported,[1,2] by affixing a polydimethylsiloxane (PDMS; SYLGARDTM 184 Silicon Elastomer Kit, DOW Co., Midland, USA) chip to a patterned nanowired Si wafer (SiNWs). The PDMS chip featured eight parallel straight microfluidic channels (length × width × height: 51 mm × 2 mm × 50 μm) with herring-bone shaped microstructures on the top wall of the

channel to generate a turbulent flow to improve contact between the substances in the blood and the SiNW wafer constituting the bottom plate.

***Macrophage membrane coating in the microfluidic device*:**

E. coli-activated, J774A.1 macrophage membranes were prepared as described before[1] by freeze-thawing and used within one day after preparation while being stored at 4°C. For macrophage membrane coating, the SiNW wafer was first coated in the microfluidic device with (3-aminopropyl) triethoxysilane (APTES, Sigma-Aldrich, Saint Louis, USA) after which bacterially-activated macrophage membrane fragments suspended in PBS were perfused through the devices.

Bacterial capture and staining:

For evaluating bacterial capture by macrophage membrane coated SiNW adsorbent surfaces in the microfluidic device, 1 mL PBS with different bacterial concentrations or with different ratios of combinations of *E. coli* and *S. aureus* were perfused through the device at a flow rate of 1 mL/h. After perfusion, the device was filled with 35 μL of a fluorescent Gram-stain (LIVE Baclight™ Bacterial Gram Stain Kit, Thermo Fisher Scientific Inc., Waltham, USA) dissolved in water (8 μM SYTO 9 and 11 μM hexidium iodide) and left for 10 min under static conditions. After staining, the adsorbent surface with captured bacteria was removed from the device, washed with PBS and examined using Confocal Laser Scanning Microscopy (Zeiss CLSM 800, Jena, Germany), operated at a magnification of 400x, yielding a field of view equal to 0.018 mm^2. Images were analyzed using Image J (National Institutes of Health, Wisconsin, USA) to calculate the number of captured Gram-stain bacteria.

Validation of bacterial absence or presence and Gram-type in bacterially-spiked human blood spiked with bacteria:

For validating the use of bacterially-activated macrophage coated SiNW adsorbent surfaces in a microfluidic device, human whole blood was obtained from healthy donors in the Soochow University Hospital with permission (January 14, 2022) from the Medical Ethics Committee (Soochow University, Suzhou, China) and the written informed consent of the donors. Blood was stored in an anticoagulant tube with ethylenediamine tetra-acetic acid (BD, New Jersey, USA) and spiked with different Gram-negative or Gram-positive bacterial strains or their combinations in concentrations ranging from above the detection limit established up to 200 CFU/mL, representing the range of clinically observed bacterial concentrations in blood of septic patients. Random bacterial concentrations and compositions of spiked blood (15 samples) were computer-generated (ChatGPT, Open AI, USA). 1 mL of each sample was perfused through a microfluidic device, as described above and CLSM images were analysed by two blinded observers, unaware of the composition of the sample, for the absence or presence of bacteria and their Gram-type.

Statistical analysis:

Data are expressed as means ± standard deviations (SD). Differences were analysed using a two-tailed Student t-test, accepting significance at $p < 0.05$ (GraphPad software package Prism 7.0, GraphPad Software, USA).

FIGURES

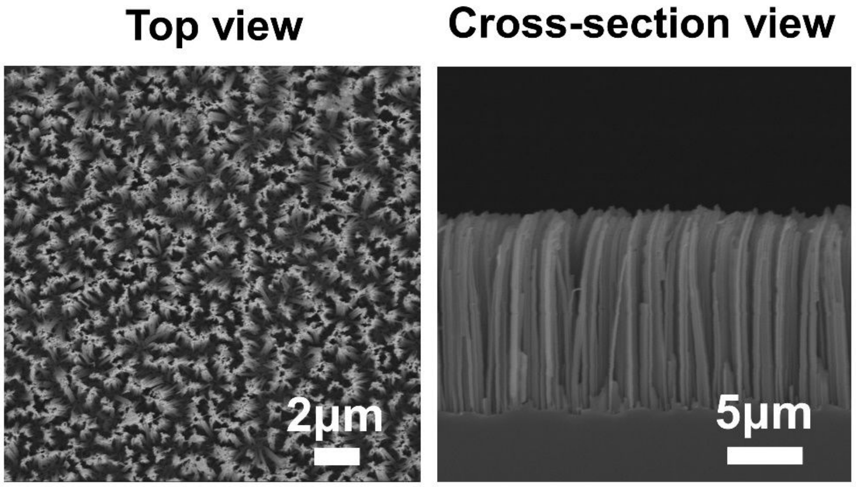

Figure S1. Scanning electron micrograph of the architecture of nanowired-Si surfaces.

REFERENCES

(1) Liu, S.; Jiang, G.; Shi, R.; Wu, R.; Xiao, X.; Yu, T.; Ren, Y.; Van der Mei, H. C.; Busscher, H. J.; Liu, J. Clearance of ESKAPE pathogens from blood using bacterially activated macrophage membrane-coated silicon nanowires. *Adv. Funct. Mater.* **2021**, 31, 2007613.

(2) Wang, S.; Liu, K.; Liu, J.; Yu, ZTF.; Xu, X.; Zhao, L.; Lee, T.; Lee, E. K.; Reiss, J.; Lee, Y. K.; Chung, L. W. K.; Huang, J.; Rettig, M.; Seligson, D.; Duraiswamy, K. N.; Shen, C. K. F. Tseng, H. R. Highly efficient capture of circulating tumor cells by using nanostructured silicon substrates with integrated chaotic micromixers. *Angew. Chem. Int. Ed. Engl.* **2011**, 50, 3084-3088.
